# Construction of a Nomogram for Predicting Survival in Elderly Patients With Lung Adenocarcinoma: A Retrospective Cohort Study

**DOI:** 10.3389/fmed.2021.680679

**Published:** 2021-07-14

**Authors:** Haisheng You, Mengmeng Teng, Chun Xia Gao, Bo Yang, Sasa Hu, Taotao Wang, Yalin Dong, Siying Chen

**Affiliations:** Department of Pharmacy, The First Affiliated Hospital of Xi'an Jiaotong University, Xi'an, China

**Keywords:** non-small-cell lung cancer, adenocarcinoma, nomogram, elderly patients, survival prediction

## Abstract

Elderly patients with non-small-cell lung cancer (NSCLC) exhibit worse reactions to anticancer treatments. Adenocarcinoma (AC) is the predominant histologic subtype of NSCLC, is diverse and heterogeneous, and shows different outcomes and responses to treatment. The aim of this study was to establish a nomogram that includes the important prognostic factors based on the Surveillance, Epidemiology, and End Results (SEER) database from 2010 to 2015. We collected 53,694 patients of older than 60 who have been diagnosed with lung AC from the SEER database. Univariate and multivariate Cox regression analyses were used to screen the independent prognostic factors, which were used to construct a nomogram for predicting survival rates in elderly AC patients. The nomogram was evaluated using the concordance index (C-index), calibration curves, net reclassification index (NRI), integrated discrimination improvement (IDI), and decision-curve analysis (DCA). Elderly AC patients were randomly divided into a training cohort and validation cohort. The nomogram model included the following 11 prognostic factors: age, sex, race, marital status, tumor site, histologic grade, American Joint Committee for Cancer (AJCC) stage, surgery status, radiotherapy status, chemotherapy status, and insurance type. The C-indexes of the training and validation cohorts for cancer-specific survival (CSS) (0.832 and 0.832, respectively) based on the nomogram model were higher than those of the AJCC model (0.777 and 0.774, respectively). The CSS discrimination performance as indicated by the AUC was better in the nomogram model than the AJCC model at 1, 3, and 5 years in both the training cohort (0.888 vs. 0.833, 0.887 vs. 0.837, and 0.876 vs. 0.830, respectively) and the validation cohort (0.890 vs. 0.832, 0.883 vs. 0.834, and 0.880 vs. 0.831, respectively). The predicted CSS probabilities showed optimal agreement with the actual observations in nomogram calibration plots. The NRI, IDI, and DCA for the 1-, 3-, and 5-year follow-up examinations verified the clinical usability and practical decision-making effects of the new model. We have developed a reliable nomogram for determining the prognosis of elderly AC patients, which demonstrated excellent discrimination and clinical usability and more accurate prognosis predictions. The nomogram may improve clinical decision-making and prognosis predictions for elderly AC patients.

## Introduction

Lung cancer is the second common cancer worldwide and the leading cause of cancer deaths (1). Non-small-cell lung cancer (NSCLC) accounts for ~85% of all lung cancer cases, with a 5-year relative survival rate of 23% (2). The elderly make up 76% of lung cancer survivors, with the median age at the diagnosis of lung cancer being 70 years (2). Age is associated with the prognosis of NSCLC patients, such as tumor recurrence and metastasis (3–5). Elderly NSCLC patients exhibit worse tolerance to surgery, radiotherapy, and chemotherapy, and therefore have worse compliance and increased side effects of anticancer treatment. The aging of organs accompanied by a decline in immune function in elderly patients increases the probability of tumor recurrence.

Adenocarcinoma (AC) is the predominant histologic subtype of NSCLC, accounting for 48.2% of cases (6). AC patients benefit from therapies targeted against specific tumor mutations (7), such as angiogenesis inhibitors, epidermal growth factor receptor inhibitors, anaplastic lymphoma kinase inhibitors, and immunotherapy drugs (2, 8, 9) but their 5-year overall survival (OS) rates remain low (2, 10). The diversity and heterogeneity of AC is related to different outcomes and responses to treatment (11–14), and so distinct therapeutic approaches and management strategies should be provided to elderly AC patients. The TNM (Tumor-Node-Metastasis) staging system was employed mainly for deciding treatment option in clinical practice. At present, the TNM (Tumor-Node-Metastasis) staging system is also a tool generally employed by oncologist for prediction tumor prognosis (15). Although TNM as a tool predicting tumor prognostication is not as common as treatment decision-making, it is a gold standard for prognostication in oncology (16). Moreover, the TNM system has several drawbacks since different factors influence the course of cancer treatment and predicting survival (16). A comprehensive prognostic prediction model therefore needs to be established, including TNM system, to more accurately predict the prognosis of patients.

Nomograms have been accepted as reliable tools for visualizing risk by incorporating and illustrating important clinical oncology factors (17). Nomograms have been demonstrated to generate more precise predictions for several types of cancer when compared with the traditional TNM staging system (18–21). The aim of this study was to establish a comprehensive prognostic evaluation model of elderly lung AC patients by constructing a nomogram that includes significant risk factors and improves AC prognoses, based on patient data from the Surveillance, Epidemiology, and End Results (SEER) database.

## Materials and Methods

### Patient Selection and Data Processing

**Figure 1:**
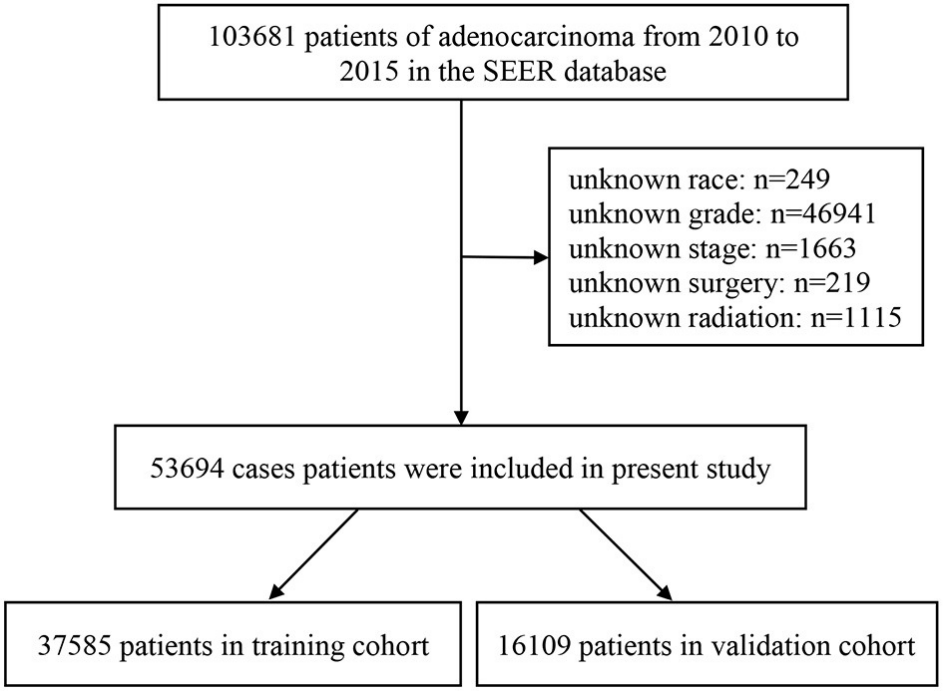
A flow diagram of patient selection process.

Patient data were extracted from the latest version of the SEER database (which covers 18 registries) using SEER^*^Stat (version 8) software. We extracted the data of patients older than 60 years who had been diagnosed with lung AC from 2010 to 2015, totaling 103,681 cases. The evaluated variables were age, sex, race, marital status, tumor site, side (lateral or bilateral), histologic grade, AJCC stage, tumor size, metastasis site, surgery status, radiotherapy status, chemotherapy status, insurance type, follow-up time, tumor-specific death, and all-cause death. Cases without data on the above variables were excluded. Our selection criteria identified 53,694 patients who met the research conditions. The selected patients were randomly divided into training and validation cohorts with a ratio of 7:3 to construct and validate the nomogram (22). Figure 1 displays a flow diagram of the patient selection process. All data were obtained free of charge from the SEER database, and this study abided by the Declaration of Helsinki and was approved by the medical ethics committee of Xi'an Jiaotong University Hospital. Informed consent was considered unnecessary for this study by the institutional review board due to its retrospective design.

### Nomogram Establishment and Statistical Analyses

Differences in the baseline characteristics between the training and validation cohorts were determined using Pearson's χ^2^ or Fisher's exact test. The variables influencing cancer-specific survival (CSS) and OS in both groups were identified using univariate and multivariate Cox proportional-hazards regression analyses. The prognostic factors identified in the multivariate analysis were used to construct the nomogram, which was tested internally and externally using the training and validation cohorts, respectively, for its ability to predict the 1-, 3-, and 5-year survival rates of NSCLC patients.

The concordance index (C-index) is the area under the receiver operating characteristic (ROC) curve (AUC) that plots the sensitivity against one minus the specificity of the nomogram. Hence, the C-index or AUC (which are often used interchangeably) for lung AC ranges from 0.5 to 1.0, with 0.5 indicating random chance and 1.0 indicating that the model was perfectly concordant with the data set. Discriminability is the accuracy in distinguishing between patients who did and did not experience an event. C-indexes and ROC curves were used to determine the discriminability of the nomogram. Calibration curves were used to evaluate the actual outcome and the predicted probability based on C-indexes. The predictive power of the model was determined using C-indexes and calibration plots. Discrimination and calibration were both evaluated using 1,000-resample bootstrapping. The net reclassification index (NRI) and integrated discrimination improvement (IDI) were measured to compare the accuracy of the nomogram with the AJCC staging model. Decision-curve analyses (DCAs) tested the clinical value of the predictive models based on their threshold probabilities. The threshold probability was used to obtain the net benefit (defined as the proportion of true positives minus the proportion of false positives, weighted by the relative harm of false-positive, and false-negative results).

All statistical analyses were performed using SPSS or R software, with *P* ≤ 0.05 indicating statistical significance.

## Results

### Patient Characteristics

This study included 53,694 patients older than 60 years with lung AC between 2010 and 2015. The 53,694 cases were divided into a training cohort (37,585 patients) and a validation cohort (16,109 patients) using random-split sampling with a ratio of 7:3. Patients aged 60–80 years, female patients, and white patients accounted for 80, 53, and 82% of the sample, respectively. The main tumor sites were the upper and lower lobes of the lung, and almost all of the lesions (98%) were unilateral. The tumors were mostly at histologic grades II and III, while the AJCC stages were mostly advanced (48.4%) with distant metastases (37%). The proportions of patients who received surgery, radiotherapy, and chemotherapy were 50, 30, and 35%, respectively. Most patients had medical insurance. The median survival time was 14 months (range 4–31 months). Half of the patients died during the follow-up period. Table 1 provides detailed information about the training and validation cohorts. In AJCC stage I -II patients, with increasing age, the ratio of surgical treatment gradually decreased, and that of radiotherapy gradually increased. From AJCC stage II to stage IV patients, the ratio of chemotherapy gradually reduced with age rise. Supplementary Table 1 shows treatment information for elderly patients with lung AC.

**Table 1:** Patients' demographics and clinicopathological characteristics.

**Variable**	**Total cohort, *n* (%)**	**Training cohort, *n* (%)**	**Validation cohort, *n* (%)**	***P*-value**
	53,694 (100%)	37,585 (70%)	16,109 (30%)	
**Age, years**				0.551
60–69	21,078 (39.26%)	14,730 (39.19%)	6,348 (39.41%)	
70–79	22,011 (40.99%)	15,462 (41.14%)	6,549 (40.65%)	
≥80	10,605 (19.75%)	7,393 (19.67%)	3,212 (19.94%)	
**Sex**, ***n***				0.427
Female	28,589 (53.24%)	20,054 (53.36%)	8,535 (52.98%)	
Male	25,105 (46.76%)	17,531 (46.64%)	7,574 (47.02%)	
**Race**, ***n***				0.828
White	44,185 (82.29%)	30,954 (82.36%)	13,231 (82.13%)	
Black	5,064 (9.43%)	3,516 (9.35%)	1,548 (9.61%)	
Asian or Pacific Islander	4,244 (7.90%)	2,973 (7.91%)	1,271 (7.89%)	
American Indian/Alaska Native	201 (0.37%)	142 (0.38%)	59 (0.37%)	
**Marital status**, ***n***				0.428
Married	29,172 (54.33%)	20,419 (54.33%)	8,753 (54.34%)	
Single	22,370 (41.66%)	15,686 (41.73%)	6,684 (41.49%)	
Unknown	2,152 (4.01%)	1,480 (3.94%)	672 (4.17%)	
**Tumor site**, ***n***				0.310
Main bronchus	753 (1.40%)	524 (1.39%)	229 (1.42%)	
Upper lobe	30,190 (56.23%)	21,119 (56.19%)	9,071 (56.31%)	
Middle lobe	2,660 (4.95%)	1,873 (4.98%)	787 (4.89%)	
Lower lobe	16,520 (30.77%)	11,623 (30.92%)	4,897 (30.40%)	
Overlapping lesion	526 (0.98%)	369 (0.98%)	157 (0.97%)	
NOS	3,045 (5.67%)	2,077 (5.53%)	968 (6.01%)	
**Lateral**, ***n***				0.336
One side	52,957 (98.63%)	37,081 (98.66%)	15,876 (98.55%)	
Bilateral	737 (1.37%)	504 (1.34%)	233 (1.45%)	
**Grade**, ***n***				0.066
I	10,366 (19.31%)	7,284 (19.38%)	3,082 (19.13%)	
II	21,484 (40.01%)	15,126 (40.24%)	6,358 (39.47%)	
III	21,411 (39.88%)	14,864 (39.55%)	6,547 (40.64%)	
IV	433 (0.81%)	311 (0.83%)	122 (0.76%)	
**AJCC Stage**, ***n***				0.376
I	21,592 (40.21%)	15,155 (40.32%)	6,437 (39.96%)	
II	6,114 (11.39%)	4,238 (11.28%)	1,876 (11.65%)	
III	8,650 (16.11%)	6,096 (16.22%)	2,554 (15.85%)	
IV	17,338 (32.29%)	12,096 (32.18%)	5242 (32.54%)	
**Tumor size**, ***n***				0.396
≤ 3 cm	27,429 (51.08%)	19,296 (51.34%)	8,133 (50.49%)	
3–5 cm	12,525 (23.33%)	8,752 (23.29%)	3,773 (23.42%)	
5–7 cm	4,891 (9.11%)	3,391 (9.02%)	1,500 (9.31%)	
≥7 cm	4,309 (8.03%)	2,986 (7.94%)	1,323 (8.21%)	
Unknown	4,540 (8.46%)	3,160 (8.41%)	1,380 (8.57%)	
**Bone metastasis**, ***n***				0.922
Yes	6,272 (11.68%)	4,404 (11.72%)	1,868 (11.60%)	
No	46,731 (87.03%)	32,697 (86.99%)	14,034 (87.12%)	
Unknown	691 (1.29%)	484 (1.29%)	207 (1.28%)	
**Brain metastasis**, ***n***	14 (4–31)	14 (4–31)		0.224
Yes	4,388 (8.17%)	3,023 (8.04%)	1,365 (8.47%)	
No	48,558 (90.43%)	34,032 (90.55%)	14,526 (90.17%)	
Unknown	748 (1.39%)	530 (1.41%)	218 (1.35%)	
**Liver metastasis**, ***n***				0.396
Yes	2,246 (4.18%)	1,583 (4.21%)	663 (4.12%)	
No	50,634 (94.30%)	35,416 (94.23%)	15,218 (94.47%)	
Unknown	814 (1.52%)	586 (1.56%)	228 (1.42%)	
**Lung metastasis**, ***n***				0.623
Yes	5,828 (10.85%)	4,050 (10.78%)	1,778 (11.04%)	
No	47,018 (87.57%)	32,946 (87.66%)	14,072 (87.35%)	
Unknown	848 (1.58%)	589 (1.57%)	259 (1.61%)	
**Surgery**, ***n***				0.142
Yes	27,305 (50.85%)	19,191 (51.06%)	8,114 (50.37%)	
No	26,389 (49.15%)	18,394 (48.94%)	7,995 (49.63%)	
**Radiation**, ***n***				0.363
Yes	15,612 (29.08%)	10,972 (29.19%)	4,640 (28.80%)	
No	38,082 (70.92%)	26,613 (70.81%)	11,469 (71.20%)	
**Chemotherapy**, ***n***				0.211
Yes	18,625 (34.69%)	12,974 (34.52%)	5,651 (35.08%)	
No	35,069 (65.31%)	24,611 (65.48%)	10,458 (64.92%)	
**Insurance**, ***n***				0.209
Yes	48,031 (89.45%)	33,662 (89.56%)	14,369 (89.20%)	
No	5,663 (10.55%)	3,923 (10.44%)	1,740 (10.80%)	
**Vital status**, ***n***				0.140
Alive	27,190 (50.64%)	19,111 (50.85%)	8,079 (50.15%)	
Dead	26,504 (49.36%)	18,474 (49.15%)	8,030 (49.85%)	
Median follow-up time (Months, 25–75th percentile)	14 (4–31)	14 (4–31)	13 (4–31)	0.525

*AJCC, the American joint committee for cancer; NOS, not otherwise specified lung cancer*.

### Prognostic Factors for CSS and OS of Elderly AC Patients

The univariate and multivariate Cox proportional-hazards regression analyses selected 11 prognostic factors for screening in the training cohort. Among these factors, a higher risk of CSS in AC patients was associated with age at diagnosis (70–79 years, HR = 1.115, *P* < 0.001; ≥80 years, HR = 1.261, *P* < 0.001), male sex (HR = 1.354, *P* < 0.001), histologic grade (II, HR = 1.372, *P* < 0.001; III, HR = 1.921, *P* < 0.001; and IV, HR = 1.818, *P* < 0.001), AJCC stage (II, HR = 2.637, *P* < 0.001; III, HR = 4.318, *P* < 0.001; and IV, HR = 8.141, *P* < 0.001), no surgery (HR = 2.833, *P* < 0.001), and no chemotherapy (HR = 1.877, *P* < 0.001), while the risk was lower for Asian or Pacific Islander race (HR = 0.749, *P* < 0.001) and tumor sites of the upper lobe (HR = 0.697, *P* < 0.001), middle lobe (HR = 0.709, *P* < 0.001), lower lobe (HR = 0.752, *P* < 0.001), and no otherwise specified lung cancer (NOS) (HR = 0.812, *P* < 0.001) (Table 2). Supplementary Table 2 lists the prognostic factors associated with OS in elderly AC patients.

**Table 2:** Cox regression analysis based on all variables for cancer-specific survival (Training Cohort).

**Characteristics**	**Univariate analysis**		**Multivariate analysis**	
	**HR (95% Cl)**	***P*-value**	**HR (95% Cl)**	***P*-value**
**Age, years**				
60–69	Reference		Reference	
70–79	1.061 (1.022–1.101)	0.002	1.115 (1.073–1.157)	<0.001
≥80	1.494 (1.432–1.560)	<0.001	1.261 (1.205–1.320)	<0.001
**Sex**, ***n***				
Female	Reference		Reference	
Male	1.418 (1.373–1.465)	<0.001	1.354 (1.309–1.401)	<0.001
**Race**, ***n***				
White	Reference		Reference	
Black	1.182 (1.119–1.247)	<0.001	0.936 (0.886–0.989)	0.019
Asian or Pacific Islander	0.905 (0.850–0.964)	0.002	0.749 (0.703–0.798)	<0.001
American Indian/Alaska Native	1.402 (1.103–1.783)	0.006	1.070 (0.841–1.361)	0.582
**Marital status**, ***n***				
Married	Reference		Reference	
Single	1.142 (1.105–1.181)	<0.001	1.093 (1.055–1.132)	<0.001
Unknown	0.938 (0.859–1.026)	0.161	0.970 (0.887–1.060)	0.500
**Primary Site**, ***n***				
Main bronchus	Reference		Reference	
Upper lobe	0.290 (0.262–0.322)	<0.001	0.697 (0.628–0.773)	<0.001
Middle lobe	0.280 (0.246–0.317)	<0.001	0.709 (0.625–0.805)	<0.001
Lower lobe	0.288 (0.259–0.320)	<0.001	0.752 (0.677–0.836)	<0.001
Overlapping lesion	0.452 (0.379–0.538)	<0.001	0.948 (0.795–1.130)	0.553
NOS	0.763 (0.680–0.855)	<0.001	0.812 (0.722–0.913)	<0.001
**Lateral**, ***n***				
One side	Reference		Reference	
Bilateral	3.178 (2.851–3.542)	<0.001	0.949 (0.842–1.070)	0.395
**Grade**, ***n***				
I	Reference		Reference	
II	1.582 (1.496–1.673)	<0.001	1.372 (1.296–1.451)	<0.001
III	3.466 (3.286–3.656)	<0.001	1.921 (1.818–2.029)	<0.001
IV	3.295 (2.809–3.866)	<0.001	1.818 (1.549–2.135)	<0.001
**AJCC Stage**, ***n***				
I	Reference		Reference	
II	2.411 (2.246–2.588)	<0.001	2.637 (2.454–2.834)	<0.001
III	4.966 (4.690–5.257)	<0.001	4.318 (4.053–4.601)	<0.001
IV	12.774 (12.154–13.427)	<0.001	8.141 (7.654–8.658)	<0.001
**Surgery**, ***n***				
Yes	Reference		Reference	
No	6.566 (6.314–6.828)	<0.001	2.833 (2.692–2.982)	<0.001
**Radiation**, ***n***				
Yes	Reference		Reference	
No	0.530 (0.513–0.548)	<0.001	1.084 (1.045–1.124)	<0.001
**Chemotherapy**, ***n***				
Yes	Reference		Reference	
No	0.588 (0.569–0.607)	<0.001	1.877 (1.809–1.948)	<0.001
**Insurance**, ***n***				
Insured	Reference		Reference	
Uninsured	1.312 (1.247–1.380)	<0.001	1.092 (1.037–1.151)	<0.001

*AJCC, the American joint committee for cancer; HR, hazard ratio; NOS, not otherwise specified lung cancer*.

### Nomogram Construction

**Figure 2:**
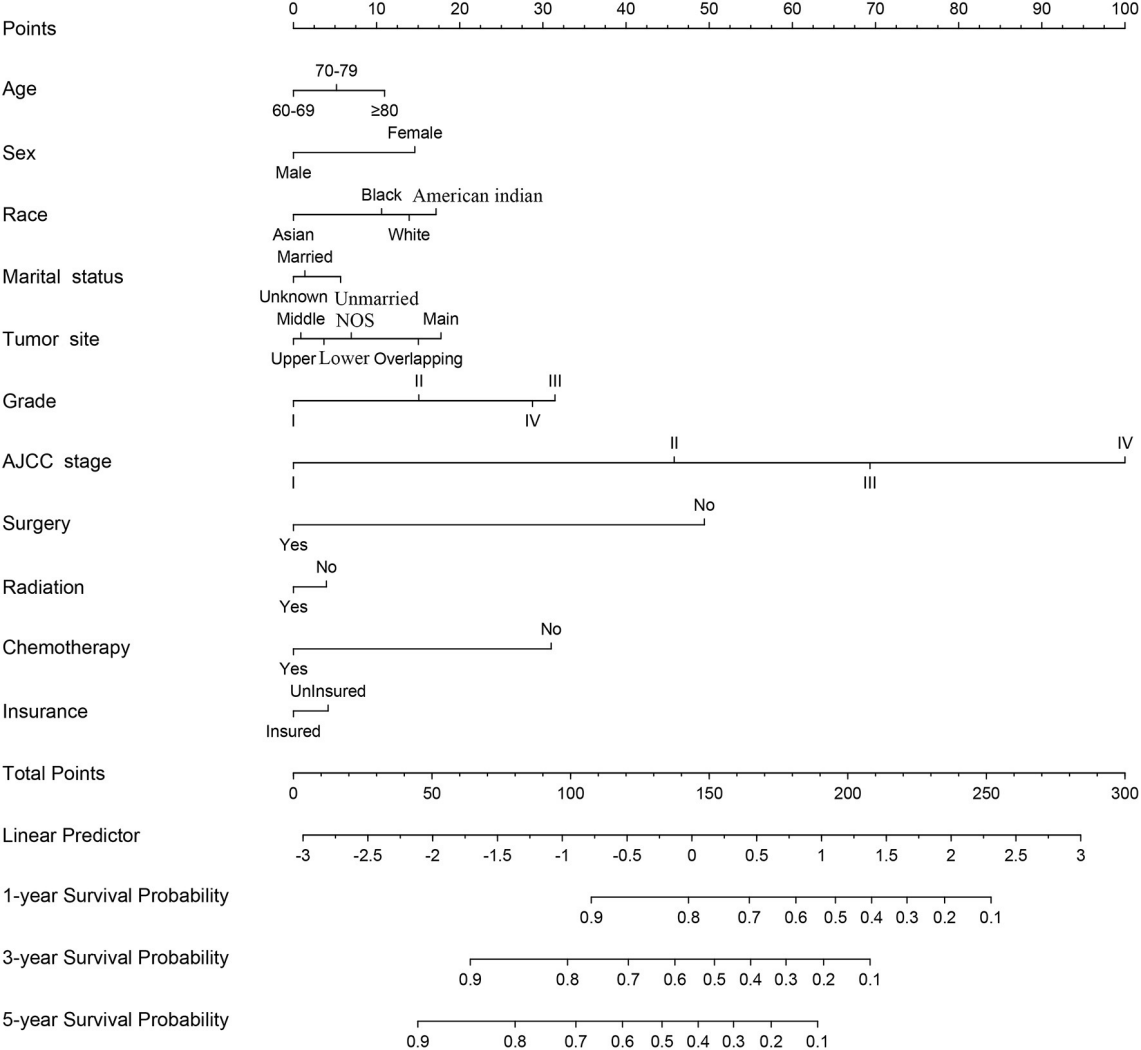
Nomogram predicted 1-, 3-, and 5-year lung adenocarcinoma cancer-specific survival for patients with 11 available factors, including age, sex, race, marital status, tumor site, grade, AJCC stage, surgery, radiation, chemotherapy, and insurance. AJCC, the American Joint Committee for Cancer; NOS, not otherwise specified lung cancer.

**Figure 3:**
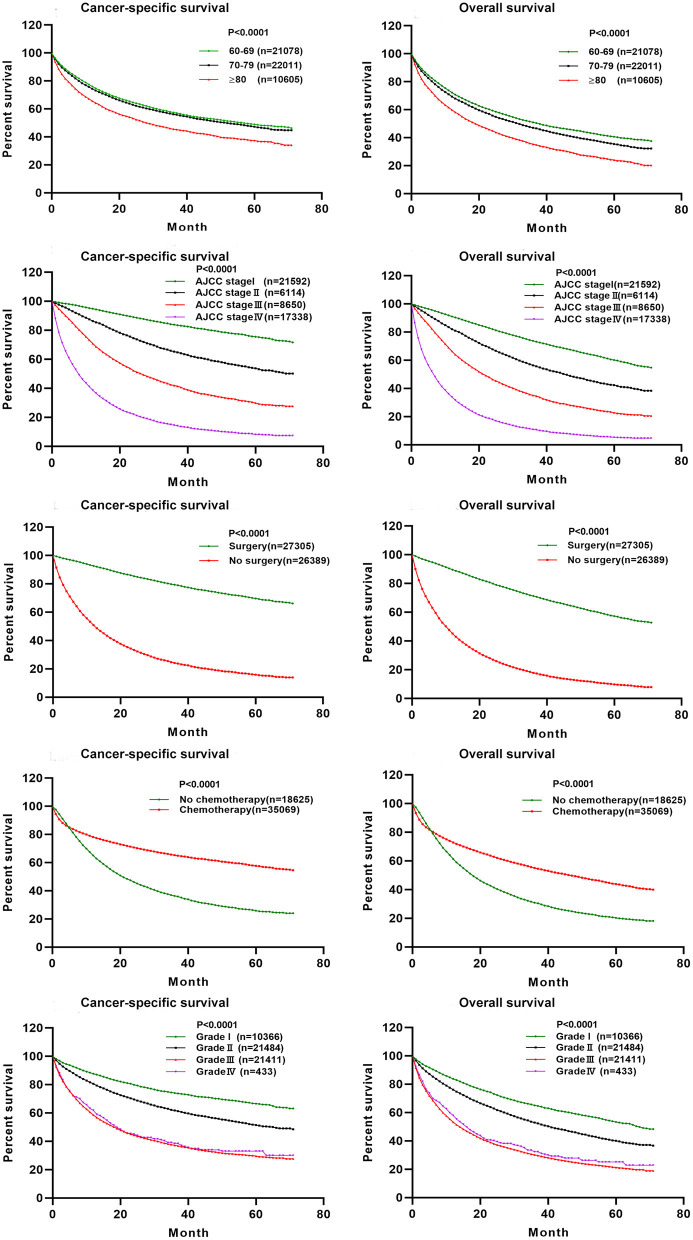
The effect of AJCC staging, surgical treatment, chemotherapy treatment, histologic grade, and age at diagnosis on the cancer-specific survival and overall survival of elderly patients with lung adenocarcinoma. Kaplan–Meier curves for cancer-specific survival (*P* < 0.001) and overall survival (*P* < 0.001).

A nomogram was constructed for predicting the 1-, 3-, and 5-year CSS of lung AC patients according to the prognostic factors selected from the training cohort (Figure 2). The CSS nomogram indicated that AJCC stage was the strongest prognostic factor, followed by surgery status, histologic grade, and chemotherapy status with a greater impact on nomogram. Patients in AJCC stages I and II, or who received surgery, or who received chemotherapy had longer CSS and OS (Figure 3). Other significant prognostic factors were tumor site, race, sex, age, marital status, radiotherapy status, and insurance type. Patients older than 80 years at diagnosis had poor CSS and OS (Figure 3).

Each level of each factor was given a score on the points scale of the nomogram. The final risk score was calculated by the sum of the score of each selected factor using the nomogram, as depicted in Figure 2, which estimated the 1-, 3-, and 5-year CSS probabilities for individual patients based on a vertical line from the total-points row. The OS nomogram was developed using the same method, as shown in Supplementary Figure 1.

### Nomogram Performance

**Figure 4:**
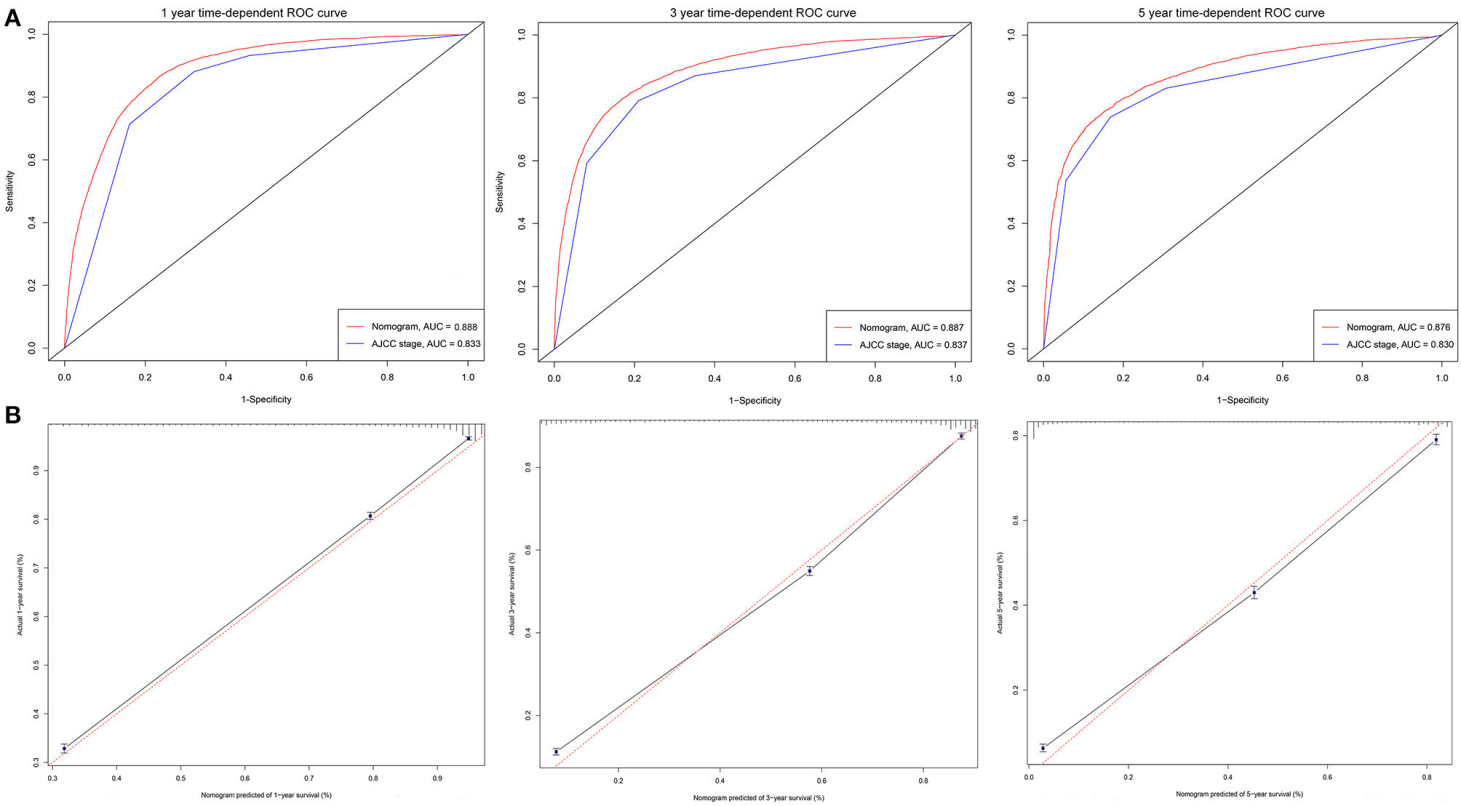
ROC curves and calibration plots for predicting patients-specific survival at 1-, 3-, and 5-year in the training cohorts. **(A)** ROC curves of the Nomogram and AJCC stage in prediction of prognosis at 1-, 3-, and 5-year point in the training set. **(B)** The calibration plots for predicting patient survival at 1-, 3-, and 5-year point in the training set. ROC, receiver operating characteristic curve; AUC, areas under the ROC curve.

**Figure 5:**
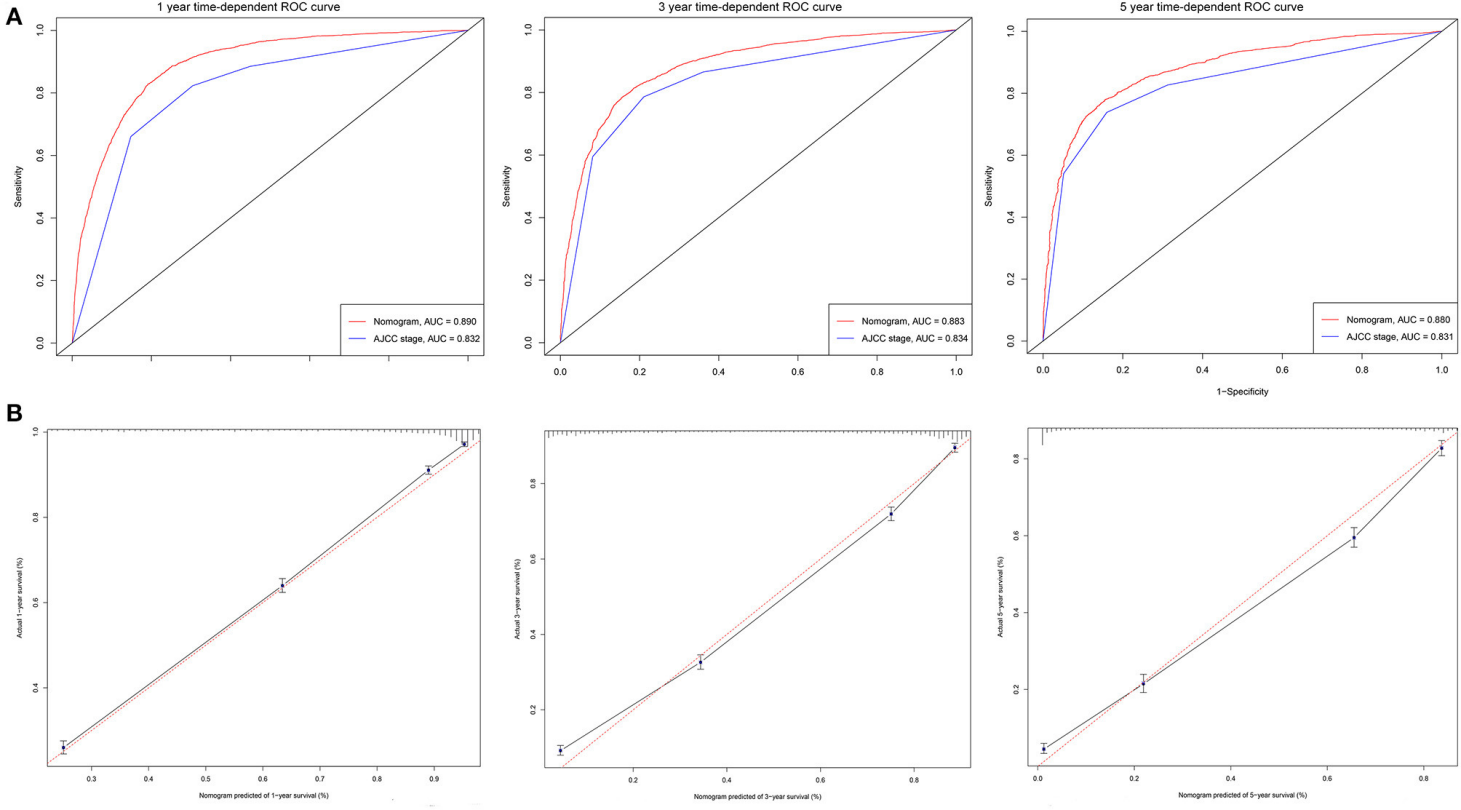
ROC curves and calibration plots for predicting patients-specific survival at 1-, 3-, and 5-year in the validation cohorts. **(A)** ROC curves of the Nomogram and AJCC stage in prediction of prognosis at 1-, 3-, and 5-year point in the validation cohorts. **(B)** The calibration plots for predicting patient survival at 1-, 3-, and 5-year point in the validation cohorts. ROC, receiver operating characteristic curve; AUC, areas under the ROC curve.

The C-indexes [nomogram C-indexes >0.70 indicate a high predictive accuracy for CSS (23)] were higher for the nomogram model (0.832 and 0.832 in the training and validation cohorts, respectively) than the AJCC staging model (0.777 and 0.774, respectively). The CSS discrimination performance as indicated by the AUC was better in the nomogram model than the AJCC staging model at 1, 3, and 5 years in both the training cohort [0.888 vs. 0.833, 0.887 vs. 0.837, and 0.876 vs. 0.830, respectively (Figure 4)] and the validation cohort [0.890 vs. 0.832, 0.883 vs. 0.834, and 0.880 vs. 0.831, respectively (Figure 5)]. The predicted 1-, 3-, and 5-year CSS probabilities corresponded with the actual observations in both the training (Figure 4) and validation (Figure 5) cohorts in calibration plots of the nomogram. The related results for OS are shown in Supplementary Figures 2, 3.

In the training cohort, the NRI values for the 1-, 3-, and 5-year CSS follow-up examinations were 0.424 (95% CI = 0.401–0.447), 0.496 (95% CI = 0.471–0.538), and 0.294 (95% CI = 0.254–0.317), respectively. The corresponding NRI values in the validation cohort were 0.446 (95% CI = 0.400–0.496), 0.484 (95% CI = 0.426–0.562), and 0.301 (95% CI = 0.229–0.355), respectively. Similarly, the IDI values for 1-, 3-, and 5-year CSS follow-up examinations were 0.060 (*P* < 0.001), 0.050 (*P* < 0.001), and 0.040 (*P* < 0.001), respectively, in the training cohort, and 0.060 (*P* < 0.001), 0.042 (*P* < 0.001), and 0.067 (*P* < 0.001) in the validation cohort. These results indicate that our model greatly improves the accuracy of prognostic predictions over the AJCC staging model.

**Figure 6:**
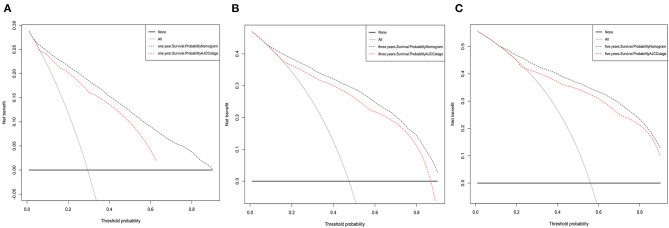
Decision curve analysis for the Nomogram and AJCC stage in prediction of prognosis of elderly lung adenocarcinoma patients at 1-year **(A)**, 3-year **(B)**, and 5-year **(C)** CSS point in the validation cohorts.

The DCAs of CSS compared the net benefits of the new model with those of the AJCC staging model. As shown in Figure 6, 1-, 3-, and 5-year outcomes of our nomogram were superior to those of the AJCC staging model across various death risk factors in the training and validation cohorts. This verifies the clinical usability and practical decision-making effects of the new model. The results of DCAs of OS are shown in Supplementary Figure 4.

## Discussion

AC is the dominant pathologic subtype of NSCLC (6, 24), and is diverse and heterogeneous. Most elderly patients have already reached an advanced cancer stage at the time of diagnosis, resulting in a poor prognosis. Although the introduction of many antitumor drugs has improved patient survival, the 5-year survival rate remains very low. TNM staging system is often applied in clinical treatment decision-making, simultaneously, is also the gold standard for survival prediction for patients (16). But TNM staging system to predict the prognosis of lung cancer patients neglects independent prognostic factors such as sex, age, histologic grade, and treatment-related factors that could improve individualized survival predictions (18). Nomograms for predicting the survival outcomes of elderly AC patients are rare. It is therefore necessary to establish a prognostic prediction model that can assist clinicians in making treatment regimens for elderly AC patients. This was the first retrospective study that we know of that used an integrated index derived from the SEER database to establish a prognostic nomogram for predicting the survival rates of elderly AC patients.

There are some unique disease characteristics for elderly AC patients. Most of them were 60–80 years old and white. The main tumor sites were the upper and lower lobes of the lung, and most lesions were unilateral. The histologic grades of tumors were mostly II and III, while the AJCC stages were mostly advanced with distant metastases. A previous study (2, 20) similarly found that 76% of lung cancer patients were old and white, more than 90% of tumors were in the upper or lower lobes of the lung, and 80–90% of AC patients among the known pathological types had poorly or moderately differentiated histologic grades (25, 26). These characteristics are similar to those in our study. Moreover, AC patients of stage I–III accounted for 67%, and ~50% received surgery in Table 1, which indicated that a higher proportion of patients with early stage adenocarcinoma undergone no surgical treatment. Elderly patients of stage I–II, from 60 to 79 years old, the surgery rates are more than 80%, while that of 80 years old or above is only 45.4% for stage I patients and 56.9% for stage II patients, respectively (Supplementary Table 1). For early stage patient surgical resection is the treatment of choice providing the best opportunity for cure and long-term survival. Still there is reluctance to recommend surgery for the elderly, partly based on the expectation that the rate of complications will be higher and elderly patients currently receive far higher rates of palliative care (27). Elderly patients are less likely to undergo curative surgery than younger patients for early-stage lung cancer although the cancer-related survival and OS are similar between older and younger patients (28). Octogenarians have poor surgery outcomes and should therefore prefer non-surgical treatments (24). 30% of the AC patients received radiotherapy in Table 1. In particular, the older and advanced patients more likely received radiation therapy (Supplementary Table 1). Stereotactic body radiation therapy is a reasonable option for high-risk surgical patients (28, 29). 35% of elderly patients with lung AC received chemotherapy in Table 1, which is lower than the data reported in the literature (30). Lung AC patients who underwent complete resection benefited from adjuvant chemotherapy (12, 31) or systemic chemotherapy with better survival (30). However, the older the patient, the less willing to receive chemotherapy (Supplementary Table 1). The median survival time was 14 months (range 4–31 months). Univariate and multivariate analyses identified 11 variables including age, sex, race, marital status, tumor site, histologic grade, AJCC stage, surgery status, radiotherapy status, chemotherapy status, and insurance type. CSS was worse in patients who had higher AJCC stages, no surgical treatment, no chemotherapy treatment, poor histologic grade, advanced age, male and single, while patients without main bronchus as the tumor site and who were Asian or Pacific Islander had longer CSS. The prognostic factors influencing OS were similar to those of CSS, which were TNM stage, no surgery, histologic grade, age, and sex.

A nomogram is a convenient graphical representation of a predictive model. This study established a new and comprehensive nomogram that combines various patient risk factors to improve prognosis predictions for elderly AC patients (Figure 2 and Supplementary Figure 1). Compared to the traditional AJCC staging model, our nomogram was capable of providing more accurate assessments and predictions for lung AC patients (Figures 4, 5). Our newly established model indicates that AJCC stage makes the greatest contribution to the prognostic score, which was similar to previous research where the 5-year overall survival rate of AC ranged from 79% for disease stage IA to 6% for stage IV (25). AC was associated with a higher risk of developing bone (32) and brain metastases (33). Our analysis indicated that surgery status, chemotherapy status, and histologic grade had greater impacts on patient survival. Surgical treatment benefits octogenarians with AC patients (34), especially for those in stage I and II (35). Chemotherapy significantly improved patient prognoses and prolonged the survival of elderly patients (30, 36). Integration of geriatric assessments can improve risk stratification and improve clinical decision-making for patients (37). Histologic grade was a significant prognostic value for patient survival, and reflects the aggressiveness of lung tumors (38, 39). In particular, grade, surgery, and chemotherapy have greatly improved the performance of nomogram. Other factors also indicated as having prognostic value include patient age, sex, race, marital status, tumor site, radiotherapy status, and insurance type. These results were consistent with previous research (24). The prognostic factors of poorly differentiated tumor grade, male sex, increased age, late stage, and patient's performance status have been shown in multiple studies to have independent negative associations with long-term survival (40, 41).

Finally, the C-index, ROC curve, and calibration curve of our model were better in the validation cohort, indicating that it provides accuracy and reliable predictions (18, 20). The significantly higher C-index of the nomogram (in both cohorts) compared with the AJCC staging model indicates the good discrimination ability of the nomogram. This indicated that the model is very precise (42). In the current study, calibration plots of predictions corresponded well with actual observations indicated by the curve being close to the 45-degree line, verifying the repeatability and reliability of the established nomogram (20, 42). This is the first nomogram constructed to predict the survival of elderly AC patients that we know of. Both physicians and their patients can use the nomogram to individualize survival predictions. We believe that the nomogram is a more precise prognostic model than the AJCC staging model and other established prognostic models.

IDI and NRI were used to evaluate the performance and clinical application of the nomogram. Compared with the AJCC staging model, the nomogram has improved accuracy and discrimination of 1-, 3-, and 5-year survival predictions for elderly AC patients. The nomogram had good discrimination and was well-calibrated, in which both IDI and NRI for 1, 3, and 5 years of follow-up examinations showed improvements in the C-index (20). DCA was also applied to compare the net benefits of the nomogram with those of the traditional AJCC staging model. Clinicians and patients can refer to the net benefit of our model according to their threshold probability during clinical decision-making. DCA values indicated that the newly established nomogram model had more practical and efficient survival predictions than the AJCC staging model (20). Our nomogram is an effective tool for predicting patient survival and optimizing treatment modalities in clinical practice.

This study was subject to several limitations. First, The SEER database does not include information on smoking history, radiotherapy doses, specific chemotherapy regimens, surgical methods, important molecular prognostic markers, comorbidity data, functional status, or other potentially important clinical information, which might reduce the predictive accuracy of the nomogram model. For example, targeted therapy and immunotherapy enhance response rates and prolong OS; The comorbidity data and functional status are most important part, closely related to the prognosis of elderly patients with lung AC. Karnofsky performance status for chemotherapy and anesthesia risk during the operation for elderly patients are important parameter in practice. Unfortunately, above information is not available in the SEER database. In the following research, these factors should be included in our model to achieve more comprehensive predictive ability for the prognosis of elderly AC patients. Second, this study was limited by collecting retrospective data from the SEER database, which may cause inherent and selection biases. The grade is also important factor of prognostic model. We screened patient data through strict inclusion and exclusion criteria. Consequently, a large number of patients without tumor grade information were excluded, which may affect the accuracy of model prediction. Finally, our nomogram is only constructed based on American patient data, and thus, may be underrepresented in the AC patients worldwide. In the following research, we would test the accuracy and generalizability of this model by external validation using Chinese patients or other populations with AC. Meanwhile, we will continue to optimize and improve this model by further clinical studies, hoping to finally have a better prognosis tool for patients with lung AC.

A nomogram for reliably determining the prognosis of elderly AC patients has been developed based on a large population sample. The nomogram includes 11 independent risk factors: AJCC stage, surgery status, chemotherapy status, histologic grade, radiotherapy status, age, sex, race, marital status, tumor site, and insurance type. Compared with the traditional AJCC staging model, the nomogram demonstrated excellent discrimination, and clinical usability, suggesting more accurate prognosis predictions for elderly AC patients. The nomogram may improve clinical decision-making as an auxiliary tool and provide accurate predictions of the prognosis of elderly AC patients.

## Data Availability Statement

The original contributions presented in the study are included in the article/Supplementary Material, further inquiries can be directed to the corresponding authors.

## Ethics Statement

All authors have signed the SEER Research Data Agreement to protect the privacy of patients, which is consistent with ethical principles.

## Author Contributions

HY, SC, and YD designed the experiments. MT, CG, BY, SH, and TW collected the data. HY and SC contributed to the statistical analysis of the data. HY wrote manuscript. All authors read and approved the final manuscript.

## Conflict of Interest

The authors declare that the research was conducted in the absence of any commercial or financial relationships that could be construed as a potential conflict of interest.
