# Supplementary material for: Construction of a Nomogram for Predicting Survival in Elderly Patients With Lung Adenocarcinoma: A Retrospective Cohort Study
Source: Front Med (Lausanne). 2021 Jul 14;8:680679. doi: 10.3389/fmed.2021.680679 (PMC8316725; doi:10.3389/fmed.2021.680679)
Supplement: Supplementary file 1 [file Data_Sheet_1.docx]

***Supplementary Material***

**Supplementary TABLE 1** The proportions of surgery, chemotherapy and radiation treatment in elderly patients with lung adenocarcinoma (Training Cohort).

|  | AJCC Stage | 60-69 years | 70-79 years | ≥80 years |
| --- | --- | --- | --- | --- |
| Surgery % |  |  |  |  |
|  | I | 89.3 | 83.3 | 45.4 |
|  | II | 86.6 | 80.1 | 56.9 |
|  | III | 47.8 | 58.7 | 30.0 |
|  | IV | 9.1 | 7.9 | 4.9 |
| Radiation % |  |  |  |  |
|  | I | 10.2 | 14.1 | 28.1 |
|  | II | 16.0 | 19.0 | 27.2 |
|  | III | 53.8 | 47.5 | 39.7 |
|  | IV | 46.6 | 40.1 | 30.1 |
| Chemotherapy % |  |  |  |  |
|  | I | 7.7 | 6.2 | 3.3 |
|  | II | 55.9 | 39.3 | 20.1 |
|  | III | 73.8 | 65.0 | 38.7 |
|  | IV | 62.5 | 52.9 | 34.9 |

AJCC, the American joint committee for cancer.

**Supplementary TABLE 2** Cox regression analysis based on all variables for overall survival (Training Cohort).

| Characteristics | Univariate analysis |  | Multivariate analysis |  |
| --- | --- | --- | --- | --- |
|  | HR(95% Cl) | *P* value | HR(95% Cl) | *P* value |
| **Age, years** |  |  |  |  |
| 60-69 | Reference |  | Reference |  |
| 70-79 | 1.125(1.088-1.163) | ＜0.001 | 1.165(1.126-1.205) | ＜0.001 |
| ≥80 | 1.598(1.539-1.660) | ＜0.001 | 1.326(1.273-1.380) | ＜0.001 |
| **Sex, n** |  |  |  |  |
| Female | Reference |  | Reference |  |
| Male | 1.447(1.406-1.490) | ＜0.001 | 1.409(1.367-1.452) | ＜0.001 |
| **Race, n** |  |  |  |  |
| White | Reference |  | Reference |  |
| Black | 1.211(1.155-1.270) | ＜0.001 | 0.976(0.930-1.025) | 0.335 |
| Asian or Pacific Islander | 0.851(0.804-0.901) | ＜0.001 | 0.716(0.675-0.759) | ＜0.001 |
| American Indian/Alaska Native | 1.313(1.053-1.638) | 0.016 | 1.028(0.824-1.282) | 0.809 |
| **Marital status, n** |  |  |  |  |
| Married | Reference |  | Reference |  |
| Single | 1.171(1.137-1.206) | ＜0.001 | 1.117(1.082-1.152) | ＜0.001 |
| Unknown | 0.968(0.895-1.047) | 0.411 | 0.989(0.914-1.070) | 0.786 |
| **Primary Site, n** |  |  |  |  |
| Main bronchus | Reference |  | Reference |  |
| Upper lobe | 0.314(0.285-0.346) |  | 0.704(0.639-0.775) | ＜0.001 |
| Middle lobe | 0.299(0.266-0.335) | ＜0.001 | 0.708(0.630-0.795) | ＜0.001 |
| Lower lobe | 0.314(0.284-0.346) | ＜0.001 | 0.758(0.687-0.836) | ＜0.001 |
| Overlapping lesion | 0.455(0.387-0.535) | ＜0.001 | 0.918(0.781-1.080) | 0.303 |
| Lung, NOS | 0.783(0.705-0.871) | ＜0.001 | 0.809(0.725-0.902) | ＜0.001 |
| **Lateral, n** |  |  |  |  |
| One side | Reference |  | Reference |  |
| Bilateral | 3.271(2.970-3.601) | ＜0.001 | 1.043(0.937-1.162) | 0.439 |
| **Grade, n** |  |  |  |  |
| I | Reference |  | Reference |  |
| II | 1.461(1.393-1.532) | ＜0.001 | 1.327(1.265-1.393) | ＜0.001 |
| III | 2.975(2.843-3.113) | ＜0.001 | 1.826(1.742-1.914) | ＜0.001 |
| IV | 2.745(2.371-3.178) | ＜0.001 | 1.691(1.460-1.959) | ＜0.001 |
| **AJCC Stage, n** |  |  |  |  |
| I | Reference |  | Reference |  |
| II | 1.854(1.749-1.966) | ＜0.001 | 2.058(1.939-2.183) | ＜0.001 |
| III | 3.453(3.295-3.619) | ＜0.001 | 3.155(2.993-3.326) | ＜0.001 |
| IV | 8.528(8.196-8.874) | ＜0.001 | 5.710(5.426-6.009) | ＜0.001 |
| **Surgery, n** |  |  |  |  |
| Yes | Reference |  | Reference |  |
| No | 5.369(5.220-5.578) | ＜0.001 | 2.744(2.624-2.869) | ＜0.001 |
| **Radiation, n** |  |  |  |  |
| Yes | Reference |  | Reference |  |
| No | 0.570(0.553-0.587) | ＜0.001 | 1.114(1.078-1.151) | ＜0.001 |
| **Chemotherapy, n** |  |  |  |  |
| Yes | Reference |  | Reference |  |
| No/Unknown | 0.684(0.665-0.705) | ＜0.001 | 1.946(1.882-2.013) | ＜0.001 |
| **Insurance, n** |  |  |  |  |
| Insured | Reference |  | Reference |  |
| UnInsured | 1.329(1.271-1.390) | ＜0.001 | 1.130(1.079-1.184) | ＜0.001 |

AJCC, the american joint committee for cancer; HR: hazard ratio; NOS, not otherwise specified lung cancer.


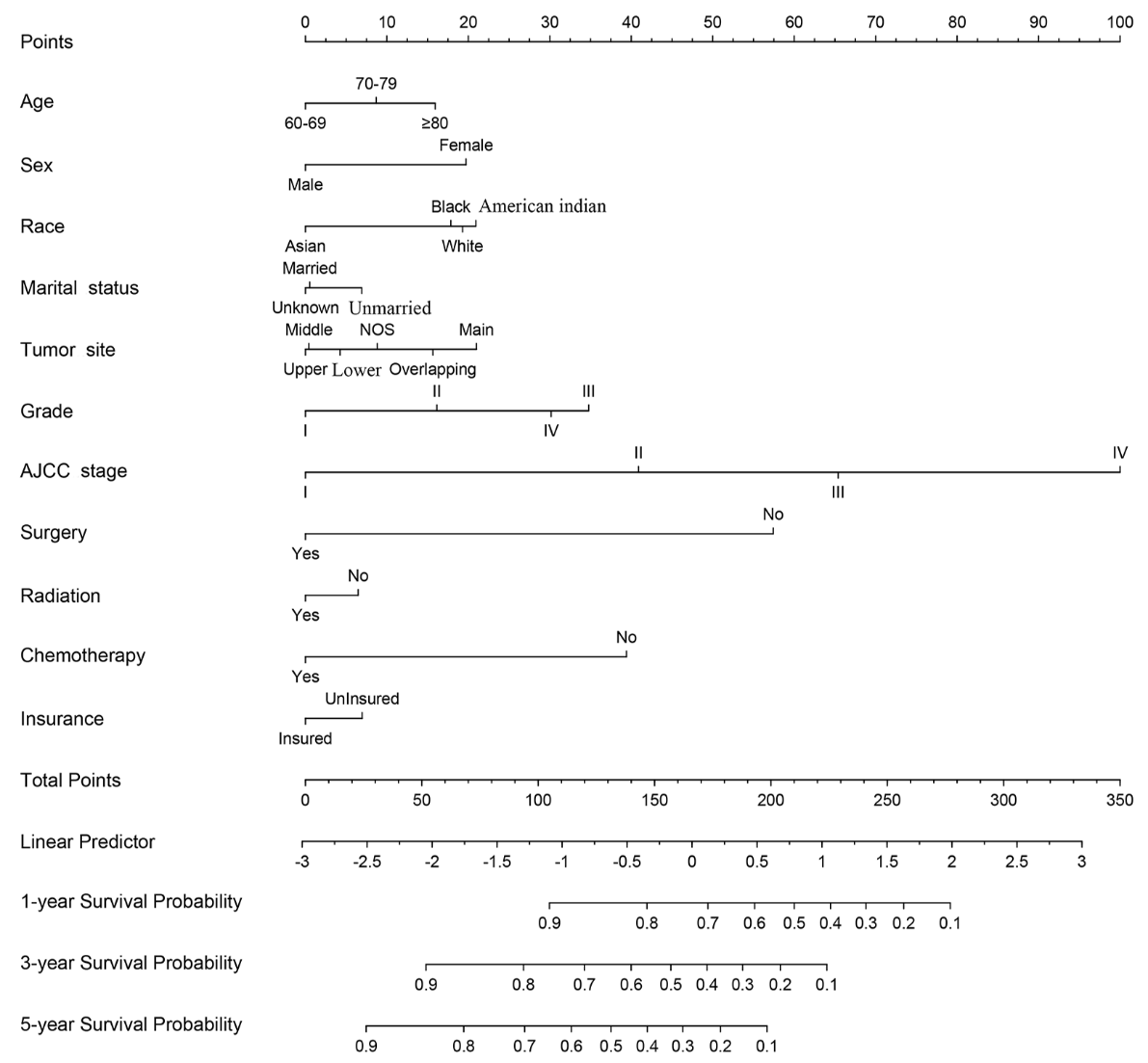


**Supplementary Figure 1**  Nomogram predicted 1-,3- and 5-year lung adenocarcinoma overall survival for patients with eleven available factors, including age, sex, race, marital status, tumor site, grade, AJCC stage, surgery, radiation, chemotherapy and insurance. AJCC, the American Joint Committee for Cancer, NOS, not otherwise specified lung cancer


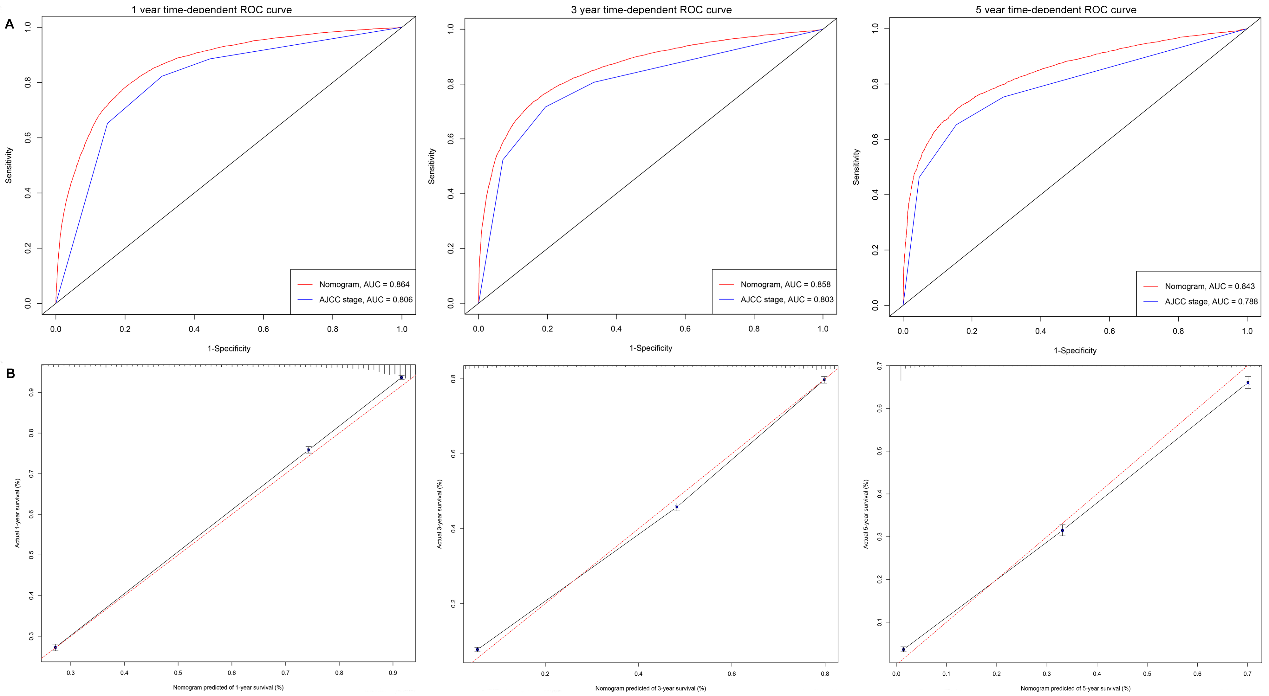


**Supplementary Figure 2** ROC curves and calibration plots for predicting patients-overall survival at 1-, 3- and 5-year in the training cohorts. (A) ROC curves of the Nomogram and AJCC stage in prediction of prognosis at 1-,3- and 5-year point in the training cohorts. (B) The calibration plots for predicting patient survival at 1-, 3- and 5-year point in the training set. ROC, receiver operating characteristic curve; AUC, areas under the ROC curve


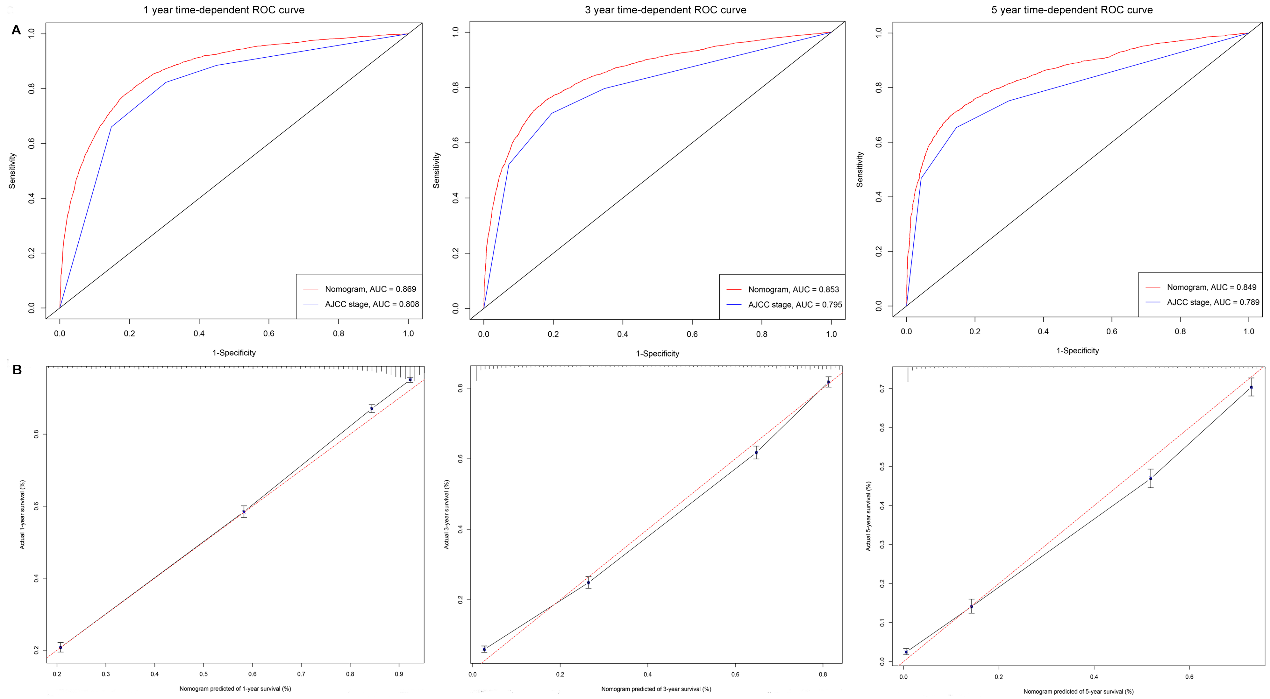


**Supplementary Figure 3** ROC curves and calibration plots for predicting patients-overall survival at 1-,3- and 5-year in the validation cohorts. (A) ROC curves of the Nomogram and AJCC stage in prediction of prognosis at 1-, 3- and 5-year point in the validation set. (B) The calibration plots for predicting patient survival at 1-, 3- and 5-year point in the validation set. ROC, receiver operating characteristic curve; AUC, areas under the ROC curve


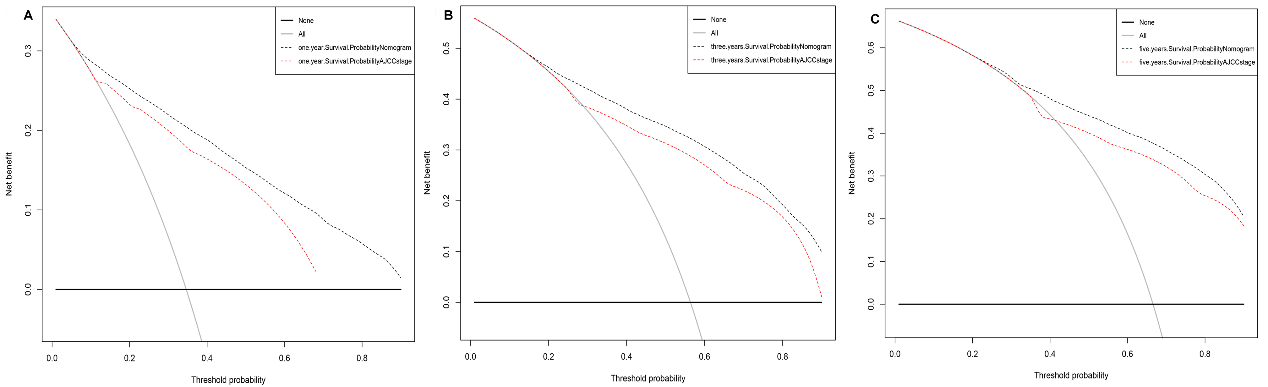


**Supplementary Figure 4** Decision curve analysis for the Nomogram and AJCC stage in prediction of prognosis of elderly lung adenocarcinoma patients at 1-year (A), 3-year (B) and 5-year (C) OS point in the validation cohorts.
